# Protocol for UVC uridine actinometry

**DOI:** 10.1016/j.mex.2022.101957

**Published:** 2022-12-26

**Authors:** Dana Pousty, Hadas Mamane, Vered Cohen-Yaniv, James R. Bolton

**Affiliations:** a School of Mechanical Engineering, Faculty of Engineering, Tel-Aviv University, Tel-Aviv, Israel; b Bolton Photosciences Inc., 628 Cheriton Cres., NW, Edmonton, AB T6R 2M5, Canada

**Keywords:** Uridine actinometry, UVC actinometry, UV-LED, UVC uridine actinometry

## Abstract

Uridine contains the chromophore uracil, a base forming part of RNA. In the range 240–290 nm, the absorption spectra of uridine and DNA are very similar and correspond to the spectral inactivation sensitivity of almost all microorganisms. This makes the uridine (absorption maximum 262 nm) an ideal actinometer for determining the germicidal photon flux in the range of 240 to 290 nm. Uridine actinometry is a simple, environmental-friendly, and easy-to-operate actinometry. Thanks to the uridine absorbance spectrum, it was found to be a perfect fit for the photon flux validation of UVC systems. Conventional UV disinfection systems are generally based on low-pressure (LP) mercury lamps which emit at 254 nm. On the other hand, UV light-emitting diodes (UV-LEDs) are a relatively new source of UV light for water treatment, emitting at various wavelengths. This protocol suggests an accurate, simple, easy to operate and straightforward way to determine the photon flux of UVC systems.

*Contain between 1 and 3 bullet points highlighting the customization rather than the steps of the procedure.*

• Because of the uridine absorbance spectrum, it is an ideal actinometer for photon flux validation of UVC systems.
• Initial uridine concentration and photoproduct absorbance impact the kinetic order and quantum yield.
• The protocol for UVC uridine actinometry is appropriate for UV-LP and UV-LED sources for water disinfection.

## Graphical abstract

**Figure:**
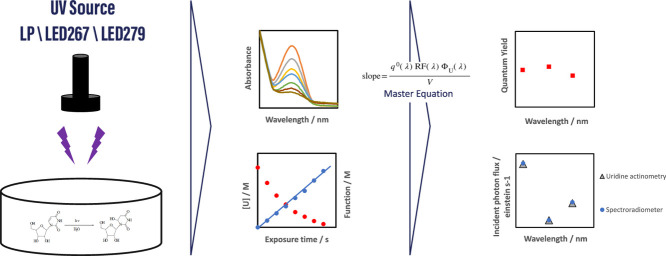

Specifications table

**Table:** 

Subject Area:	Chemistry
More specific subject area:	*UV actinometry*
Method name:	*UVC uridine actinometry*
Name and reference of original method:	Determination of the germicidal irradiance by uridine actinometry and calibration of UV sensors” by O. Hoyer and K. Nick, unpublished, about 2000
Resource availability:	*N.A.*

## Method details

#### Background

**Fig. 1:**
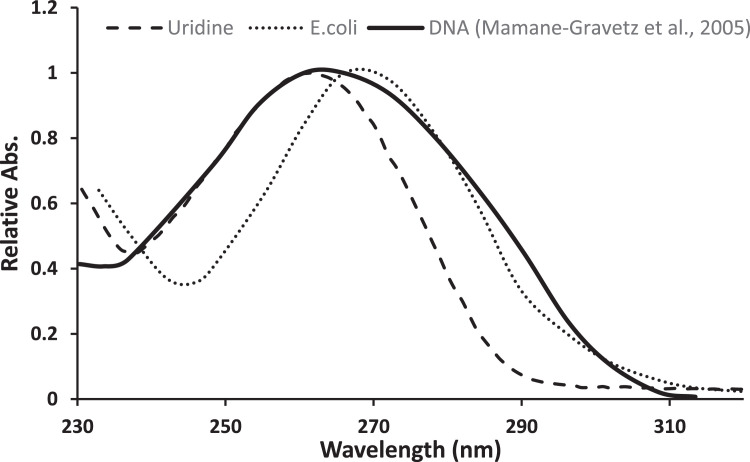
UV spectrum of uridine, the UV spectrum of DNA [10], and the action spectra of *E. coli* each spectrum normalized to its peak wavelength.

**Fig. 2:**
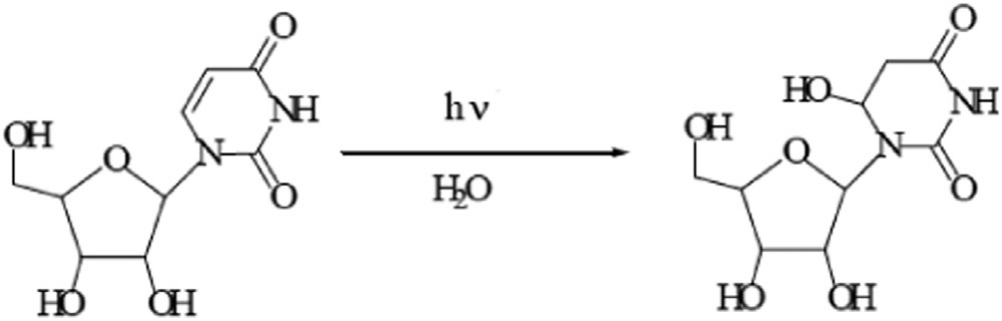
Uridine photohydrate formation after irradiation of uridine with UV light.

Chemical actinometry is a well-established method for radiation measurements in the UV range [9,13]. Chemical actinometers can be used for physical radiometer calibration [4,12] and measurement of the UV photon flux in complex photoreactors and batch photosystems. Among the well-established chemical actinometry methods, the uridine actinometer can be more applicable to measuring the germicidal photon flux in the germicidal range of 240 to 290 nm, since uridine and DNA have very similar absorption spectra and correspond to the spectral inactivation sensitivity of almost all microorganisms (Fig. 1) [5,8]. However, there are cases (e.g., MS2 coliphage and Adenovirus 2), where the spectral inactivation sensitivity differs from the DNA absorbance below 240 nm [1,7]. Thus, this uridine actinometry protocol is not recommended below 240 nm. The uridine chromophore, as uracil a base-forming part of RNA, has a maximum absorbance at 262 nm. Because of the low absorbance of the photoproduct (uridine hydrate, Fig. 2) at the 262 nm wavelength, it is possible to track the reaction spectrophotometrically.

The uridine aqueous solution (pH = 7) is photolyzed in a collimated beam apparatus in an open Petri dish (with a ring mask) [3].

The following are the key uridine parameters:

**Table:** 

Molecular weight	244.2 g mol^–1^
Molar absorption coefficients	[13]
253.7 nm	8410 M^–1^ cm^–1^
262 nm	10,185 M^–1^ cm^–1^

Quantum yield [11]

**Table:** 

253.7 nm	0.025 ± 0.004
267 nm	0.028 ± 0.002
279 nm	0.023 ± 0.002

Let [U] be the uridine concentration (M) in an aqueous solution. The equations were validated for low initial concentration and compared to the standard actinometry (ferrioxalate). The general expression for the rate of decay (M s^–1^) of uridine in a collimated beam apparatus is

(1) $$-\frac{d\left[U\right]}{\mathrm{dt}}=\;\frac{q_{p}\left(\mathrm{abs}\right)\left(\lambda \right)\Phi_{U}\left(\lambda \right)}{V}=\;\frac{q_{p}^{0}\left(\lambda \right)\mathrm{RF}\left(\lambda \right)x_{U}\left(\lambda \right)\Phi_{U}\left(\lambda \right)}{V}$$

where *q*_p_(abs)(*λ*) is the absorbed photon flux (einstein s^–1^) at wavelength *λ*

*q*_p_^0^(*λ*) is the incident photon flux.
RF(*λ*) is the reflection factor at wavelength *λ*
  $$x_{U}\left(\lambda \right)=\left[1-10^{-\varepsilon_{U}\left(\lambda \right)\left[U\right]l}\right]$$

is the fraction of light absorbed by uridine.

*l*_U_(*λ*) is the uridine molar absorption coefficient (M^–1^ cm^–1^) at wavelength *λ*
*l* is the path length (cm).
*V* is the volume (L).

Φ_U_(*λ*) is the uridine quantum yield at wavelength *λ* which does not depend on temperature [6] and is nearly independent of the wavelength [14] in the spectral range of 240–290 nm.

Bolton et. al [2] showed that Eq. (1) can be integrated analytically as follows:

(2) $$\left\{{\left[U\right]}_{0}-{\left[U\right]}_{t}\right\}+\;\frac{1}{\ln\left(10\right)\Phi_{C}\left(\lambda \right)l}\text{ln}\;\left\{\frac{1-\text{exp}\left[-\ln\left(10\right)\Phi_{U}\left(\lambda \right){\left[U\right]}_{0}l\right]}{1-\text{exp}[-\ln\left(10\right)\Phi_{U}\left(\lambda \right){\left[U\right]}_{t}l}\right\}=\frac{q^{0}\left(\lambda \right)\;\mathrm{RF}\;\left(\lambda \right)\Phi_{C}\left(\lambda \right)}{V}t$$

Hence, a plot of the left-hand side of Eq. (2) versus the time *t* in seconds should be linear with a slope

(3) $$\mathrm{slope}\;=\;\frac{q^{0}\left(\lambda \right)\mathrm{RF}\;\left(\lambda \right)\Phi_{U}\left(\lambda \right)}{V}$$

If the incident photon irradiance *E*_p_^0^(*λ*) (einstein m^–2^ s^–1^) or irradiance *E*^0^(*λ*) (W m^–2^) at the center of the Petri dish is required

(4) $$E_{p}^{0}\;\left(\lambda \right)=\frac{q^{0}\left(\lambda \right)}{PF\times A_{Mask}}\;\text{or}\;E^{0}\left(\lambda \right)=\frac{q^{0}\left(\lambda \right)\;U\left(\lambda \right)}{PF\times A_{Mask}}$$

where PF is the Petri Factor, *A*_Mask_ is the internal area (m^3^) of the hole in the ring mask on top of the Petri dish, and *U*(*λ*) (J einstein^–1^) is the energy of one einstein of photons at wavelength *λ*.

**Fig. 3:**
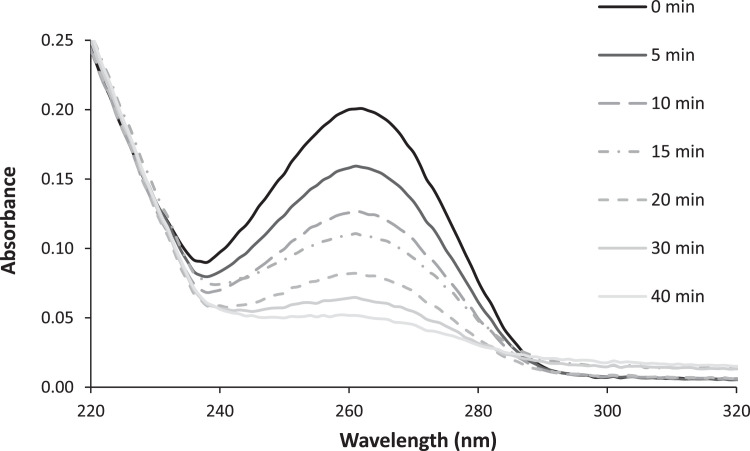
The absorption spectrum of a uridine solution at a concentration of 2.8 mM before and after a low-pressure mercury lamp irradiation of various duration (numbers on curves – irradiation times in min).

The degree of uridine conversion to uridine hydrate is determined photometrically at 262 nm (see Fig. 3).

The uridine solution absorbance is affected by the absorbance photoproduct. Hence, the following correction is required for all the uridine solution concentrations [U]*_t_* at time *t*:

(5) $${\left[U\right]}_{t}=\frac{A_{t}-\left(\varepsilon_{H}\times {\left[U\right]}_{0}\times l_{Petri}\right)}{\left(\varepsilon_{U}-\varepsilon_{H}\right)\times l_{Petri}}$$

where *A_t_* is the solution absorbance at 262 nm at time *t*, [U]_0_ is the uridine concentration at time 0, $l_{petri}$ is the Petri dish path length (cm), and ε_H_ is the photoproduct molar absorption coefficient (M^–1^ cm^–1^) at wavelength 262 nm, which is determined from the following equation:

(6) $$\varepsilon_{H}=\left(\frac{A_{\infty }}{A_{0}}\right)\varepsilon_{U}$$

where *A*_∞_ is the solution absorbance at an infinite time at 262 nm, measured made by long exposure when absorbance was stable in time, and *A*_0_ is the solution absorbance at time 0.

### Range of application

Uridine absorbs strongly in the range of 240–290 nm. The calibration with uridine actinometry is only possible when the UV lamps and sensors meet the following criteria:

• Low-pressure mercury UV.
• Light-emitting diodes lamp (UV-LED) – lamp emitting in the required uridine spectral range.

An appropriate UV lamp should be used depending on whether the sensors for low-pressure or UV-LEDs are calibrated.

#### Materials and equipment

• Laboratory absorbance scan apparatus with reference sensor according, e.g., spectroradiometer (using an Ocean Optics USB4000 spectroradiometer equipped with a cosine corrector, OCF- 104447, EOS-A1241604), or radiometer.
• Petri dishes, e.g., Disposable Petri dishes polystyrene, diameter 87 mm, Pyrex®, diameter 30 mm. Note that the Petri dishes do not have to be disposable, and the diameter may vary, this factor is considered in Eqs. (3) and (4).
• 100 mL volumetric flask.
• Micropipettes with disposable tips, e.g., 50, 100, 200, 10,000 µL.
• Spectrophotometer for measurements in the spectral range 200–400 nm, with a spectral resolution Δ*λ* = 2 nm and a sensitivity of 0.1 mAU (Thermo Scientific Evolution 220 double beam UV-Vis spectrophotometer, THS840-210600S).
• 5 cm quartz cuvette.

### Solutions

All reagents must be analytical grade.

#### Solution A – water - double-distilled, free of UV absorbing substances

The absence of these substances is assessed by recording the spectrum from 200 to 400 nm. The absorbance *A* must be less than 0.002 at 254 and 262 nm determined in a 5 cm cuvettes.

#### Solution B – phosphate buffer (1 mM), pH 7.0

(2.5 ± 0.05) mL of a colorless standard buffer solution (commercial product: phosphate buffer solution pH 6.88, about 0.04 M) is made up to 100 mL with water (solution A). A sufficiently low absorbance is tested as described above.

#### Solution C – uridine stock solution, 7.4 ± 0.2 mM in water

180 ± 5 mg uridine (Uridine ≥99% Sigma-Aldrich, U370, CAS: 58-96-8 molecular weight 244.2 g mol^-1^) is dissolved in 100 mL water (solution A). The stock solution can be used for about one week when kept in a dark refrigerator (with an aluminum foil cover) at 4°C.

#### Experimental procedure

##### Uridine calibration test solution in buffer

The uridine concentration must be adjusted to exhibit an absorbance of ≤ 0.1 at 262 nm for the path length to be used for the irradiation (as shown in Table 1). In a 100 mL volumetric flask, a corresponding volume of uridine stock solution according to Table 1 is added with a microliter pipette and thoroughly mixed and filled up to the mark with 1 mM buffer (solution B).

**Table 1:** Uridine concentrations and corresponding irradiation path lengths that give an absorbance of 0.1 or 85 % transmittance at 262 nm

Parameter	Units	Values			
Irradiation path length *d*	cm	0.5*	1.0	1.5	2.0
Concentration *c*_o_	*µ*M	14	7	4.6	3.5
Volume of stock solution added to 100 mL test solution	*µ*L	200	100	65	50

⁎ 30 mL uridine solution in an 87 mm Petri dish (surface area 60 cm^2^); the irradiation path length is 0.5 cm – the initial concentration of the test solution should therefore be 14 µM uridine. 100 mL 1 mM buffer solution is, therefore, spiked with 200 µL stock solution and thoroughly mixed.

The quality of the test solution is controlled by recording the spectrum from 200 to 400 nm in a 5 cm cuvette (Fig. 3). This value represents the initial value of the absorbance *A*_o_ at 262 nm.

The precision of uridine actinometry decreases when the uridine concentration increases [6], hence, the uridine concentration in the irradiation solution should be kept low. However, is not stable and must be prepared fresh from the stock for each calibration test right before the experiment.

#### Uridine actinometry experiment

The UV exposure is performed in a collimated beam apparatus with a low-pressure UV lamp, or a UV-LED lamp at the required spectral range, and the temperature of the test solution must be between 20 and 25 °C.

1. The UV lamp should be switched on for about 30 min to allow thermal stabilization.
2. The distance between the UV lamp and the Petri dish that holds the sample solution is adjusted so that the irradiance is about 1.5 W/m^2^ (0.15 mW/cm^2^) and homogeneous across the Petri dish less than ± 2% deviation. A UV sensor (such as radiometer or spectroradiometer) should be positioned to scan over the Petri dish every 5 mm in the x and y directions, in order to verify less than 2% variation, as described by Bolton and Linden [3].
3. Before and after the actinometry experiment, position the radiometer/spectroradiometer detector head so that the “calibration plane” (not necessarily the top of the detector head – this plane should be specified in the calibration certificate for the measurement device) is at the level of the top of the washer/ring mask or cardboard plate, and record the measurement device irradiance value.
4. The incident photon flux [*q*_0_(253.7) (einstein s^–1^)] is then given by
  (6) $$q\left(253.7\right)\;=\;E\left(253.7\right)\times \text{PF}/\left(0.1196266/\left(253.7\times 10^{-9}\right)\right)$$

where *E* (253.7) is the irradiance (W m^–2^) at the center of the hole in the ring mask that is measured with the radiometer or spectroradiometer, the wavelength is according to the peak wavelength of the UV source [11],

*A* is the area (m^2^) of the hole in the ring mask

The factor 0.1196266 is *hcN*_A_

PF is the Petri Factor which measured according to [3]

5. For the irradiation, four equal time intervals are chosen to cover the irradiation period *t*_mid_ necessary for a >50% drop in uridine concentration. The irradiation time *t*_mid_ can be calculated from the estimated irradiance *E*_estim_ in W/m² and the factor *F* = 6.97 × 10 J/m^2^ (factor *F* is defined below):
  $$t_{\text{max}}=F/E_{\text{estim}}=6.97\times 10/E_{\text{stim}}\;\left(\text{sec}\right)$$

For instance, the maximum irradiation time at an irradiance of 13.82 W/m^2^ should be about 963 s. During this time, minimum three irradiation runs are performed at equal intervals (e.g., 5, 10, and 15 min).

6. The usual procedure is to stop the irradiation after (e.g., 30 min) by closing the shutter. A portion of the solution is transferred with a pipette into a dry quartz cuvette with a 5 cm optical path, and the absorbance is measured in a laboratory spectrophotometer at 262 nm. The solution is transferred back to the Petri dish, allowing only minor remnants to adhere to the cuvette walls (should be less than 250 µL and can be determined by weighing the cuvette). The irradiation is then continued for another 30 min, and the subsequent photometric measurement is carried out in the same way. The procedure is repeated through to the last interval.

The photometric determinations yield values for the absorbance *A*_o_ before the irradiation and the absorbance values *A*_1_, *A*_2_, *A*_3_ after the irradiation periods.

### Calculations

The uridine concentrations (M) at various exposure times should be determined using the uridine molar absorption coefficient ε_U_(262) = 10,185 M^–1^ cm^–1^. These concentrations should then be inserted into the function on the left-hand side of Eq. (2) and plotted against the exposure time in seconds. The slope is then Eq. (3). If the photon flux *q*_0_(253.7) is known, the quantum yield Φ_U_(253.7) can be determined and vice versa [2].

**Fig. 4:**
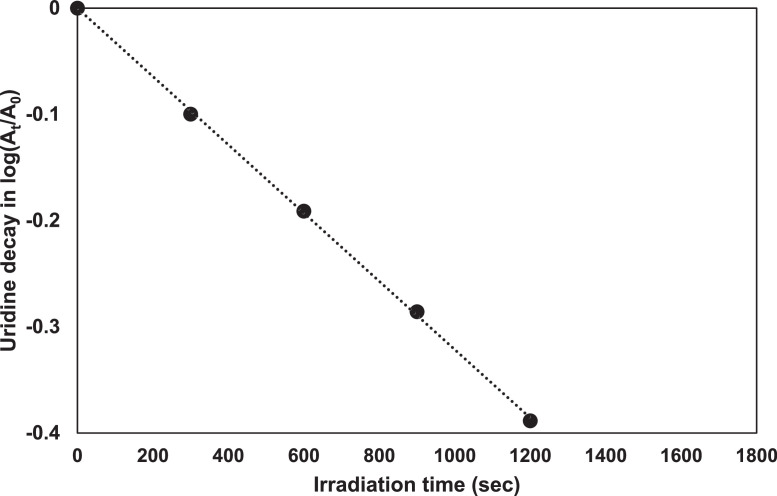
A typical plot of the left-hand side of Eq. (2) versus exposure time.

This procedure was developed for exposures using a low-pressure UV lamp (monochromatic at 253.7 nm). A new effective UV source, UV-LED, is becoming widely used for water treatment. As most common actinometry methods are calibrated for the traditional UV mercury lamps, it is required to conduct adaptations and optimization to fit the new UV source which exhibits different light characteristics. Use of a monochromatic assumption for a UV-LED using the center wavelength has been compared to a process using numerical integration over the emission spectrum of the UV-LED. A simplification of the calculations has been examined by [11] using this protocol. No significant differences were observed between the monochromatic analysis and the polychromatic analysis, measured by the calibrated spectroradiometer and the ferrioxalate actinometer. Hence, with UV-LEDs, monochromatic analysis using only the peak wavelength of the UV-LED can be used Fig. 4.

Determination of the irradiation time *t*_50_ for 50% decomposition. The data log(*A*_t_/*A*_o_) is plotted versus irradiation time. The intercept of the regression line with the abscissa at log(*A*_t_/*A*_o_) = –0.3 gives the value of *t*_50_ (in this case 963 s), which corresponds to an irradiance *E* of 13.82 W/m^2^.

**Additional information:** We also give you the option to submit both supplementary material and additional information. Supplementary material relates directly to the work that you have submitted and can include extensive excel tables, raw data etc. We would also encourage you to include failed methods or describe adjustments to your methods that did not work. Additional information can include anything else that is not directly related to your method, e.g. more general background information, useful links etc. Introduction is not a section included in the MethodsX format. This information could be moved to the end under Additional Information.

## Declaration of Competing Interest

The authors declare that they have no known competing financial interests or personal relationships that could have appeared to influence the work reported in this paper.

## Data Availability

Data will be made available on request.
